# Fractional diffusion on the human proteome as an alternative to the multi-organ damage of SARS-CoV-2

**DOI:** 10.1063/5.0015626

**Published:** 2020-08-11

**Authors:** Ernesto Estrada

**Affiliations:** Instituto Universitario de Matemáticas y Aplicaciones, Universidad de Zaragoza, 50009 Zaragoza, Spain and ARAID Foundation, Government of Aragón, 50018 Zaragoza, Spain

## Abstract

The coronavirus 2019 (COVID-19) respiratory disease is caused by the novel coronavirus SARS-CoV-2 (severe acute respiratory syndrome coronavirus 2), which uses the enzyme ACE2 to enter human cells. This disease is characterized by important damage at a multi-organ level, partially due to the abundant expression of ACE2 in practically all human tissues. However, not every organ in which ACE2 is abundant is affected by SARS-CoV-2, which suggests the existence of other multi-organ routes for transmitting the perturbations produced by the virus. We consider here diffusive processes through the protein–protein interaction (PPI) network of proteins targeted by SARS-CoV-2 as an alternative route. We found a subdiffusive regime that allows the propagation of virus perturbations through the PPI network at a significant rate. By following the main subdiffusive routes across the PPI network, we identify proteins mainly expressed in the heart, cerebral cortex, thymus, testis, lymph node, kidney, among others of the organs reported to be affected by COVID-19.

Severe acute respiratory syndrome coronavirus 2 (SARS-CoV-2) is the new coronavirus causing the pandemic known as coronavirus disease 2019 (COVID-19). This respiratory disease is characterized by multi-organ and systemic damages in patients. The abundance of ACE2 on human organs has been claimed as responsible for such multi-organ spread of the virus damages. However, once on circulation, the virus could spread to practically every organ in the human body as ACE2 is ubiquitous on endothelia and smooth muscle cells of virtually all organs. Contrastingly, SARS-CoV-2 only damages selectively a few organs. Here, we develop the hypothesis that the effects of the SARS-CoV-2 virus can be spread through the human protein–protein interaction (PPI) network in a subdiffusive way. We then elaborate a time-fractional diffusion model on networks, which allow us to study this phenomenon. Starting the diffusion from the SARS-CoV-2 spike protein to the human PPI network, we show here that the perturbations can spread across the whole network in a very few steps. Consequently, we discover a few potential routes of propagation of these perturbations from proteins mainly expressed in the lungs to proteins mainly expressed in other different tissues, such as the heart, cerebral cortex, thymus, lymph node, testis, prostate, liver, small intestine, duodenum, kidney, among others already reported as damaged by COVID-19.

## I. INTRODUCTION

Since December 2019, a new coronavirus designated SARS-CoV-2 (severe acute respiratory syndrome coronavirus 2)^1^ has produced an outbreak of pulmonary disease, which has soon become a global pandemic.^2,3^ The new coronavirus 2019 (COVID-19) disease is characterized by a wide range of clinical manifestations,^4,5^ with an important implication of multi-organ and systemic dysfunctions,^6,7^ which include heart failure, renal failure, liver damage, shock, and multi-organ failure. The new coronavirus shares about 82% of its genome with the one that produced the 2003 outbreak (SARS-CoV).^8^ Both coronaviruses share the same cellular receptor, which is the angiotensin-converting enzyme 2 (ACE2) receptor.^8–10^ ACE2 receptors are enriched in several tissues across the organs, such as in alveolar epithelial type II cells of lung tissues, in the heart, endothelium, kidneys, and intestines. Therefore, ACE2 has been hypothesized as a potential cause of the major complications of the COVID-19.^11,12^ However, it has been found that ACE2 has abundant expression on endothelia and smooth muscle cells of virtually all organs.^13^ Therefore, it should be expected that after SARS-CoV-2 is present in circulation, it can be spread across all organs. In contrast, both SARS-CoV and SARS-CoV-2 are found specifically in some organs but not in others, as shown by *in situ* hybridization studies for SARS-CoV. This was already remarked by Hamming *et al.*^13^ by stressing that it “is remarkable that so few organs become viruspositive, despite the presence of ACE2 on the endothelia of all organs and SARS-CoV in blood plasma of infected individuals.” Recently, Gordon *et al.*^14^ identified human proteins that interact physically with those of the SARS-CoV-2 forming a high confidence SARS-CoV-2-human protein–protein interaction (PPI) system. Using this information, Gysi *et al.*^15^ discovered that 208 of the human proteins targeted by SARS-CoV-2 forms a connected component inside the human PPI network. That is, these 208 are not randomly distributed across the human proteome, but they are closely interconnected by short routes that allow moving from one to another in just a few steps. These interdependencies of protein–protein interactions are known to enable that perturbations on one interaction propagate across the network and affect other interactions.^16–19^ In fact, it has been signified that diseases are a consequence of such perturbation propagation.^20–22^ It has been stressed that the protein–protein interaction process requires diffusion in their initial stages.^23^ The diffusive processes occur when proteins, possibly guided by electrostatic interactions, need to encounter each other many times before forming an intermediate.^24^ Not surprisingly, diffusive processes have guided several biologically oriented searches in PPI networks.^25,26^ Therefore, we assume here that perturbations produced by SARS-CoV-2 proteins on the human PPI network are propagated by means of diffusive processes. However, due to the crowded nature of the intra-cell space and the presence in it of spatial barriers, subdiffusive processes more than normal diffusion are expected for these protein–protein encounters.^27–29^ This creates another difficulty, as remarked by Batada *et al.*,^23^ which is that such (sub)diffusive processes along are not sufficient for carrying out cellular processes at a significant rate in cells.

Here, we propose the use of a time-fractional diffusion model on the PPI network of proteins targeted by SARS-CoV-2. The goal is to model the propagation of the perturbations produced by the interactions of human proteins with those of SARS-CoV-2 through the whole PPI. The subdiffusive process emerging from the application of this model to the SARS-CoV-2-human PPIs has a very small rate of convergence to the steady state. However, this process produces a dramatic increment of the probability that certain proteins are perturbed at very short times. This kind of shock wave effect of the transmission of perturbations occurs at much earlier times in the subdiffusive regime than at the normal diffusion one. Therefore, we propose here a switch and restart process in which a subdiffusive process starts at a given protein of the PPI, perturbs a few others, which then become the starting point of a new subdiffusive process and so on. Using this approach, we then analyze how the initial interaction of the SARS-CoV-2 spike protein with a human protein propagates across the whole network. We discover some potential routes of propagation of these perturbations from proteins mainly expressed in the lungs to proteins mainly expressed in other different tissues, such as the heart, cerebral cortex, thymus, lymph node, testis, prostate, liver, small intestine, duodenum, kidney, among others.

## II. MATERIALS AND METHODS

### A. Settling a model

The problem we intend to model here is of a large complexity as it deals with the propagation of perturbations across a network of interacting proteins, each of which is located in a crowded intra-cellular space. Therefore, we necessarily have to impose restrictions and make assumptions to settle our modeling framework. As we have mentioned in Sec. I, protein encounters should necessarily occur in subdiffusive ways due to the crowded environment in which they are embedded, as well as the existence of immobile obstacles such as membranes. By a subdiffusive process, we understand that the mean square displacement of a protein scales as

$$\left\langle x^{2}\left(t\right)\right\rangle ∼t^{\kappa },$$ (1)

where 0<κ<1 is the anomalous diffusion exponent. As observed by Sposini *et al.*,^30^ these anomalous diffusive processes can emerge from (i) continuous time random walk (CTRW) processes or by (ii) viscoelastic diffusion processes. In the first case, the “anomaly” is created by power-law waiting times in between motion events. This kind of processes is mainly accounted for by the generalized Langevin equation with the power-law friction kernel as well as by fractional Brownian motion (FBM). While the first processes are characterized by the stretched Gaussian displacement probability density, weak ergodicity, and aging, the second ones are ergodic processes characterized by the Gaussian density probability distribution. Therefore, our first task is to discern which of these two kinds of approaches is appropriate for the current scenario.

We start by mentioning that Weiss *et al.*^31^ have analyzed data from fluorescence correlation spectroscopy (FCS) for studying subdiffusive biological processes. They have, for instance, reported that membrane proteins move subdiffusively in the endoplasmic reticulum and Golgi apparatus *in vivo*. Subdiffusion of cytoplasmatic macromolecules was also reported by Weiss *et al.*^32^ using FCS. Then, Guigas and Weiss^27^ simulated the way in which the subdiffusive motion of these particles should occur in a crowded intracellular fluid. They did so by assigning diffusive steps from a Weirstrass–Mandelbrot function yielding a FBM. They stated that CTRW was excluded due to its Markovian nature. In another work, Szymanski and Weiss^33^ used FCS and simulations to analyze the subdiffusive motion of a protein in a simulated crowded medium. First, they reported that crowded-induced subdiffusion is consistent with the predictions from FBM or obstructed (percolation-like) diffusion. Second, they reported that CTRW does not explain the experimental results obtained by FCS and should not be appropriated for such processes.

The time resolution of FCS is in the microsecond range, i.e., $10^{-6}$ s.^34^ However, an important question on biological subdiffusion may require higher time resolution to be solved. This is the question of how diffusive processes on short times, while the macromolecule has not felt yet the crowding of the environment, is related to the long-time diffusion. This particular problem was explored experimentally by Gupta *et al.*^35^ by using state-of-the-art neutron spin-echo (NSE) and small-angle neutron scattering (SANS), which has a resolution in the nanosecond range, i.e., $10^{-9}$ s. Their experimental setting was defined by the use of two globular proteins in a crowded environment formed by poly(ethylene oxide) (PEO), which mimics a macromolecular environment. In their experiments, NSE was used to tackle the fast diffusion process, which corresponds to a dynamics inside a trap built by the environment mesh. SANS captures the slow dynamics, which corresponds to the long-time diffusion at macroscopic length scales. From our current perspective, the most important result of this work is that the authors found that in a higher concentration of polymeric solutions, like in the intra-cellular space, the diffusion is fractional in nature. They showed this by using the fractional Fokker–Planck equation with a periodic potential. According to Gupta *et al.*,^35^ this fractional nature of the crossover from fast dynamics to slow macroscopic dynamics is due to the heterogeneity of the polymer mesh in the bulk sample, which may well resemble the intra-cellular environment. As proved by Barkai *et al.*,^36^ the fractional Fokker–Planck equation can be derived from the CTRW, which clearly indicates that the results obtained by Gupta *et al.* point out to the classification of the subdiffusive dynamics into the class (i). We should remark that independently of these results by Gupta *et al.*,^35^ Shorten and Sneyd^37^ have successfully used the fractional diffusion equation to mimic the protein diffusion in an obstructed media like within skeletal muscle. We notice in passing that the (fractional) diffusion equation can be obtained from the (fractional) Fokker–Planck equation in the absence of an external force.

In closing, because here we are interested in modeling the diffusion of proteins in several human cells, which are highly crowded, and in which we should recover the same crossover between initial fast and later slow dynamics, we will consider a modeling tool of the class (i). In particular, we will focus our modeling on the use of a time-fractional diffusion equation using Caputo derivatives. Another justification for the use of this model here is that interacting proteins can be in different kinds of cells. Thus, we consider that the perturbation of one protein is not necessarily followed by the perturbation of one of its interactors, but a time may mediate between the two processes. This is exactly the kind of processes that the time-fractional diffusion captures.

### B. Time-fractional diffusion model on networks

In this work, we always consider G=(V,E) to be an undirected finite network with vertices V representing proteins and edges E representing the interaction between pairs of proteins. Let us consider 0<α≤1 and a function u:[0,∞)→R, then we denote by $D_{t}^{\alpha }u$ the fractional Caputo derivative of u of the order α, which is given by^38^

$$D_{t}^{\alpha }u\left(t\right)=\int_{0}^{t}g_{1-\alpha }\left(t-\tau \right)u^{\prime }\left(\tau \right)\;d\tau :=\left(g_{1-\alpha }*u^{\prime }\right)\left(t\right),\;t>0,$$

where ∗ denotes the classical convolution product on (0,∞) and $g_{\gamma }\left(t\right)\frac{t^{\gamma -1}}{\Gamma (\gamma )},$ for γ>0,where Γ(⋅) is the Euler gamma function. Observe that the previous fractional derivative has sense whenever the function is derivable and the convolution is defined (for example, if $u^{\prime }$ is locally integrable). The notation $g_{\gamma }$ is very useful in the fractional calculus theory, mainly by the property $g_{\gamma }*g_{\delta }=g_{\gamma +\delta }$ for all γ,δ>0.

Here, we propose to consider the time-fractional diffusion (TFD) equation on the network as

$$D_{t}^{\alpha }x\left(t\right)=-CLx\left(t\right),$$ (2)

with the initial condition $x\left(0\right)=x_{0}$, where $x_{i}\left(t\right)$ is the probability that the protein i is perturbed at the time t; C is the diffusion coefficient of the network, which we will set hereafter to unity; and L is the graph Laplacian, i.e., L=K−A, where K is a diagonal matrix of node degrees and A is the adjacency matrix. This model was previously studied in distributed coordination algorithms for the consensus of multi-agent systems.^39–41^ The use of fractional calculus in the context of physical anomalous diffusion has been reviewed by Metzler and Klafter.^42^ A different approach has been developed by Riascos and Mateos.^43,44^ It is based on the use of fractional powers of the graph Laplacian (see Ref. 45 and references therein). The approach has been recently formalized by Benzi *et al.*^46^ This method cannot be used in the current framework because it generates only superdiffusive behaviors (see Benzi *et al.*^46^) and not subdiffusive regimes. Another disadvantage of this approach is that it can only be used to positive (semi)definite graph operators, such as the Laplacian, but not to adjacency operators such as the one used in tight-binding quantum mechanical or epidemiological approaches (see Sec. VI).

Theorem 1 The solution of the fractional-time diffusion model on the network is

$$x\left(t\right)=E_{\alpha ,1}\left(-{\left(tC\right)}^{\alpha }L\right)x_{0},$$ (3)

where $E_{\alpha ,\beta }\left(\gamma L\right)$ is the Mittag–Leffler function of the Laplacian matrix of a graph.

Proof. We use the spectral decomposition of the network Laplacian $L=U\Lambda U^{-1}$, where $U=\left[{\vec{\psi }}_{1}\cdots {\vec{\psi }}_{n}\right]$ and $\Lambda =diag\left(\mu_{r}\right)$. Then, we can write

$$D_{t}^{\alpha }x\left(t\right)=-U\Lambda U^{-1}x\left(t\right).$$ (4)

Let us define $y\left(t\right)=U^{-1}x\left(t\right)$ such that $D_{t}^{\alpha }x\left(t\right)=-U\Lambda y\left(t\right)$, and we have

$$\begin{array}{rl} U^{-1}D_{t}^{\alpha }x\left(t\right) & =-\Lambda y\left(t\right),\\ D_{t}^{\alpha }y\left(t\right) & =-\Lambda y\left(t\right). \end{array}$$ (5)

As Λ is a diagonal matrix, we can write

$$D_{t}^{\alpha }y_{i}\left(t\right)=-\mu_{i}y_{i}\left(t\right),$$ (6)

which has the solution

$$y_{i}\left(t\right)=E_{\alpha ,1}\left(-t^{\alpha }\mu_{i}\right)y_{i}\left(0\right).$$ (7)

We can replace $y_{i}\left(t\right)=U^{-1}x_{i}\left(t\right)$ to have

$$\begin{array}{rl} U^{-1}x_{i}\left(t\right) & =E_{\alpha ,1}\left(-t^{\alpha }\mu_{i}\right)U^{-1}x_{i}\left(0\right),\\ x_{i}\left(t\right) & =UE_{\alpha ,1}\left(-t^{\alpha }\mu_{i}\right)U^{-1}x_{i}\left(0\right), \end{array}$$ (8)

which finally gives the result in the matrix-vector when written for all the nodes,

$$x\left(t\right)=E_{\alpha ,1}\left(-t^{\alpha }L\right)x_{0}.$$ (9)

We can write $L=U\Lambda U^{-1}$, where $U=\left[{\vec{\psi }}_{1}\cdots {\vec{\psi }}_{n}\right]$ and $\Lambda =diag\left(\mu_{r}\right)$. Then,

$$E_{\alpha ,1}\left(-t^{\alpha }L\right)=UE_{\alpha ,1}\left(-t^{\alpha }\Lambda \right)U^{-1},$$ (10)

which can be expanded as

$$\begin{array}{rl} E_{\alpha ,1}\left(-t^{\alpha }L\right) & ={\vec{\psi }}_{1}{\vec{\phi }}_{1}^{T}E_{\alpha ,1}\left(-t^{\alpha }\mu_{1}\right)+{\vec{\psi }}_{2}{\vec{\phi }}_{2}^{T}E_{\alpha ,1}\left(-t^{\alpha }\mu_{2}\right)\\ & \;+\cdots +{\vec{\psi }}_{n}{\vec{\phi }}_{n}^{T}E_{\alpha ,1}\left(-t^{\alpha }\mu_{n}\right), \end{array}$$ (11)

where ${\vec{\psi }}_{j}$ and ${\vec{\phi }}_{j}$ are the jth column of U and of $U^{-1}$, respectively. Because $\mu_{1}=0$ and $0<\mu_{2}\leq \cdots \leq \mu_{n}$ for a connected graph, we have

$$\lim_{t\to \infty }E_{\alpha ,1}\left(-t^{\alpha }L\right)={\vec{\psi }}_{1}{\vec{\phi }}_{1}^{T},$$ (12)

where ${\vec{\psi }}_{1}^{T}{\vec{\phi }}_{1}=1$. Let us take ${\vec{\psi }}_{1}=\vec{1}$, such that we have

$$\lim_{t\to \infty }\vec{x}\left(t\right)=\lim_{t\to \infty }\left(E_{\alpha ,1}\left(-t^{\alpha }L\right)\right){\vec{x}}_{0}=\left(\vec{1}{\vec{\phi }}_{1}^{T}\right){\vec{x}}_{0}=\left({\vec{\phi }}_{1}^{T}{\vec{x}}_{0}\right)\vec{1}.$$ (13)

This result indicates that in an undirected and connected network, the diffusive process controlled by the TFD equation always reaches a steady state, which consists of the average of the values of the initial condition. In the case of directed networks (PPI are not directed by nature) or in disconnected networks (a situation that can be found in PPIs), the steady state is reached in each (strongly) connected component of the graph. Also, because the network is connected, $\mu_{2}$ makes the largest contribution to $E_{\alpha ,1}\left(-t^{\alpha }L\right)$ among all the nontrivial eigenvalues of L. Therefore, it dictates the rate of convergence of the diffusion process. We remark that in practice, the steady state $\lim_{t\to \infty }\left\|x_{v}\left(t\right)-x_{w}\left(t\right)\right\|=0,\forall v,w\in V$ is very difficult to achieve. Therefore, we use a threshold ε, e.g., $\varepsilon =10^{-3},$ such that $\lim_{t\to \infty }\left\|x_{v}\left(t\right)-x_{w}\left(t\right)\right\|=\varepsilon$ is achieved in a relatively small simulation time.

Due to its importance in this work, we remark the structural meaning of the Mittag–Leffler function of the Laplacian matrix appearing in the solution of the TFD equation. That is, $E_{\alpha ,1}\left(-t^{\alpha }L\right)$ is a matrix function, which is defined as

$$E_{\alpha ,1}\left(-t^{\alpha }L\right)=\sum_{k=0}^{\infty }\frac{{\left(-t^{\alpha }L\right)}^{k}}{\Gamma \left(\alpha k+1\right)},\alpha >0,$$ (14)

where Γ(⋅) is the Euler gamma function as before. We remark that for α=1, we recover the diffusion equation on the network: dx(t)/dt=−Lx(t) and its solution $E_{1,1}\left(-t^{\alpha }L\right)=\exp\left(-tCL\right)$ is the well-known heat kernel of the graph.

### C. Time-fractional diffusion distance

We define here a generalization of the diffusion distance studied by Coifman and Lafon.^47^ Let $f\left(\tau L\right)=E_{\alpha ,1}\left(-t^{\alpha }L\right)$, such that $f{\left(\tau L\right)}_{vw}$ represents the v,w entry of the matrix function $E_{\alpha ,1}\left(-t^{\alpha }L\right)$. Then, we define the following quantity:

$$D_{vw}=f{\left(\tau L\right)}_{vv}+f{\left(\tau L\right)}_{ww}-2f{\left(\tau L\right)}_{vw}.$$ (15)

We have the following result.

Theorem 2 The function $D_{vw}=f{\left(\tau L\right)}_{vv}+f{\left(\tau L\right)}_{ww}-2f{\left(\tau L\right)}_{vw}$ is a Euclidean distance between the corresponding pair of nodes in the network.

Proof. The matrix function f(τL) can be written as $f\left(\tau L\right)=Uf\left(\tau \Lambda \right)U^{-1}$. Let ${\vec{\varphi }}_{u}={\left[\psi_{1,u},\psi_{2,u},\ldots ,\psi_{n,u}\right]}^{T}$. Then,

$$D_{vw}={\left({\vec{\varphi }}_{v}-{\vec{\varphi }}_{w}\right)}^{T}f\left(\tau \Lambda \right)\left({\vec{\varphi }}_{v}-{\vec{\varphi }}_{w}\right).$$ (16)

Therefore, because f(τL) is positively defined, we can write

$$\begin{array}{rl} D_{vw} & =\left({\left({\vec{\varphi }}_{v}-{\vec{\varphi }}_{w}\right)}^{T}f^{1/2}\left(\tau \Lambda \right)\right)\left(f^{1/2}\left(\tau \Lambda \right)\left({\vec{\varphi }}_{v}-{\vec{\varphi }}_{w}\right)\right)\\ & ={\left(f^{1/2}\left(\tau \Lambda \right){\vec{\varphi }}_{v}-f^{1/2}\left(\tau \Lambda \right){\vec{\varphi }}_{w}\right)}^{T}\left(f^{1/2}\left(\tau \Lambda \right){\vec{\varphi }}_{v}-f^{1/2}\left(\tau \Lambda \right){\vec{\varphi }}_{w}\right)\\ & ={\left({\vec{x}}_{v}\left(\tau \right)-{\vec{x}}_{w}\left(\tau \right)\right)}^{T}\left({\vec{x}}_{v}\left(\tau \right)-{\vec{x}}_{w}\left(\tau \right)\right)\\ & ={\left\|{\vec{x}}_{v}\left(\tau \right)-{\vec{x}}_{w}\left(\tau \right)\right\|}^{2}, \end{array}$$ (17)

where ${\vec{x}}_{v}\left(\tau \right)=f^{1/2}\left(\tau \Lambda \right){\vec{\varphi }}_{v}$. Consequently, $D_{vw}$ is a square Euclidean distance between v and w. In this sense, the vector ${\vec{x}}_{v}\left(\tau \right)=f^{1/2}\left(\tau \Lambda \right){\vec{\varphi }}_{v}$ is the position vector of the node v in the diffusion Euclidean space.

Because $E_{1,1}\left(-t^{\alpha }L\right)=\exp\left(-t^{\alpha }L\right)$, we have that $D_{vw}$ generalizes the diffusion distance studied by Coifman and Lafon, which is the particular case when α=1. Let $\mu_{j}$ be the jth eigenvalue and $\psi_{ju}$ the uth entry of the jth eigenvector of the Laplacian matrix. Then, we can write the time-fractional diffusion distance as

$$D_{vw}=\sum_{j=1}^{n}{\left(\psi_{ju}-\psi_{jv}\right)}^{2}E_{\alpha ,1}\left(-\tau \mu_{j}\right).$$ (18)

It is evident that when α=1, $D_{vw}$ is exactly the diffusion distance previously studied by Coifman and Lafon.^47^ The fractional-time diffusion distance between every pair of nodes in a network can be represented in a matrix form as follows:

$$\Sigma \left(\tau \right)={\left(\vec{s}\left(\tau \right){\vec{1}}^{T}+\vec{1}\vec{s}{\left(\tau \right)}^{T}-2f\left(\tau L\right)\right)}^{{}^{\circ}1/2},$$ (19)

where $\vec{s}=\left[f{\left(\tau L\right)}_{11},f{\left(\tau L\right)}_{22},\ldots ,f{\left(\tau L\right)}_{nn}\right]$ is a vector whose entries are the main diagonal terms of the Mittag–Leffler matrix function, $\vec{1}$ is an all-ones vector, and ° indicates an entrywise operation. Using this matrix, we can build the diffusion distance-weighted adjacency matrix of the network,

W(τ)=A°Σ(τ)=Σ(τ)°A. (20)

The shortest diffusion path between two nodes is then the shortest weighted path in W(τ).

Lemma 3 The shortest (topological) path distance between two nodes in a graph is a particular case of the time-fractional shortest diffusion path length for τ→0.

Proof. Let us consider each of the terms forming the definition of the time-fractional diffusion distance and apply the limit of the very small $-t^{\alpha }$. That is,

$$\lim_{\tau \to 0}f{\left(\tau L\right)}_{vv}=\lim_{\tau \to 0}\sum_{r=1}^{n}\psi_{rv}^{2}f\left(\tau \Lambda \right)=1,$$ (21)

and in a similar way,

$$\begin{array}{rl} \lim_{\tau \to 0}f{\left(\tau L\right)}_{vw} & =\lim_{\tau \to 0}\sum_{r=1}^{n}\psi_{rv}\psi_{rw}f\left(\tau \Lambda \right)\\ & =\sum_{r=1}^{n}\psi_{rv}\psi_{rw}=0. \end{array}$$ (22)

Therefore, $\lim_{\tau \to 0}D_{vw}\left(\tau \right)=2$. Consequently, $\lim_{\tau \to 0}W\left(\tau \right)=2A$, which immediately implies that the time-fractional shortest diffusion path is identical to the shortest (topological) one in the limit of very small $\tau =-t^{\alpha }$.

### D. Network of proteins targeted by SARS-CoV-2

The proteins of SARS-CoV-2 and their interactions with human proteins were determined experimentally by Gordon *et al.*^14^ Gysi *et al.*^15^ constructed an interaction network of all 239 human proteins targeted by SARS-CoV-2. In this network, the nodes represent human proteins targeted by SARS-CoV-2 and two nodes are connected if the corresponding proteins have been determined to interact with each other. Obviously, this network of proteins targeted by SARS-CoV-2 is a subgraph of the protein–protein interaction (PPI) network of humans. One of the surprising findings of Gysi *et al.*^15^ is the fact that this subgraph is not formed by proteins randomly distributed across the human PPI, but they form a main cluster of 208 proteins and a few small isolated components. Hereafter, we will always consider this connected component of human proteins targeted by SARS-CoV-2. This network is formed by 193 proteins, which are significantly expressed in the lungs. Gysi *et al.*^15^ reported a protein as being significantly expressed in the lungs if its GTEx median value is larger than 5. GTEx^48^ is a database containing the median gene expression from RNA-seq in different tissues. The other 15 proteins are mainly expressed in other tissues. However, in reporting here, the tissues that were proteins are mainly expressed; we use the information reported in The Human Protein Atlas^49^ where we use information not only from GTEx but also from HPA (see details at the Human Protein Atlas webpage) and FANTOM5^50^ datasets.

## III. RESULTS

### A. Global characteristics of the time-fractional diffusion

The PPI network of human proteins targeted by SARS-CoV-2 is very sparse, having 360 edges, i.e., its edge density is 0.0167, 30% of nodes have a degree (number of connections per protein) equal to one, and the maximum degree of a protein is 14. The second smallest eigenvalue of the Laplacian matrix of this network is very small; i.e., $\mu_{2}=0.0647$. Therefore, the rate of convergence to the steady state of the diffusion processes taking place on this PPI is very slow. We start by analyzing the effects of the fractional coefficient α on these diffusive dynamics. We use the normal diffusion α=1 as the reference system.

**FIG. 1.**
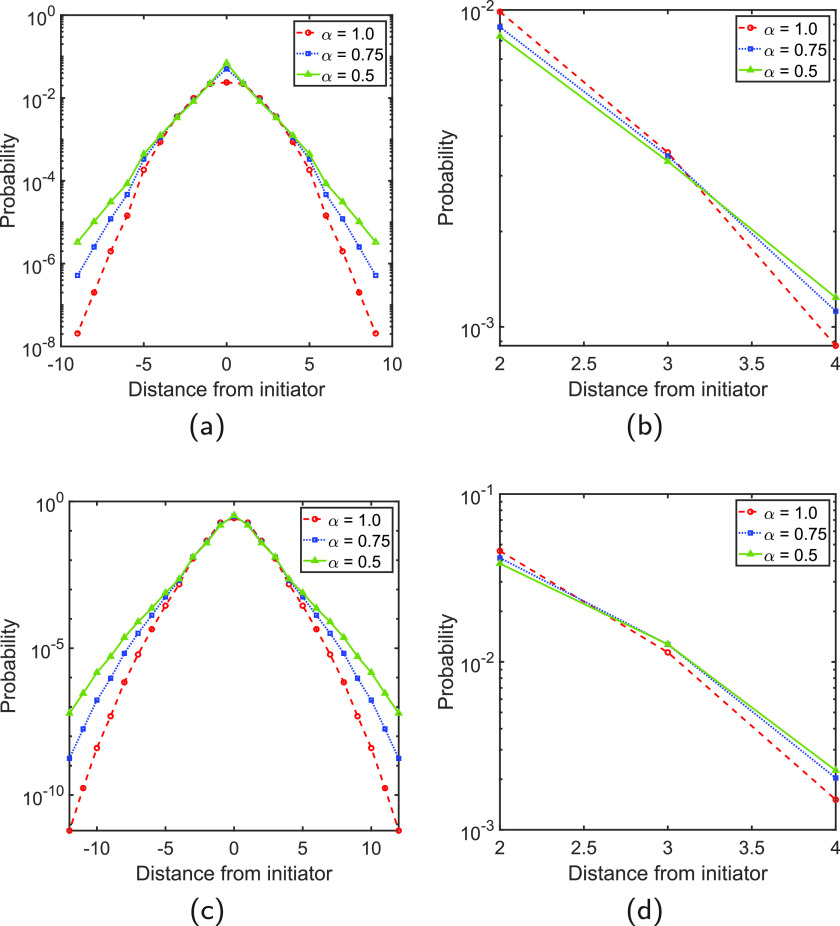
Spatial propagation of perturbations. The average probability that a protein at a given distance from the perturbed protein feels the perturbation. (a) The initiators are the protein PRKACA (a) and (b) as well as MRPS5 (c) and (d). (b) and (d) zoom the scale of the processes in (a) and (c), respectively. The y axis is in a logarithmic scale. The distance (x axis) refers to the shortest path distance from the initiator, and the plots are made “artificially” symmetric for better visualization.

To analyze the effects of changing α over the diffusive dynamics on the PPI network, we consider the solution of the TFD equation for processes starting at a protein with a large degree, i.e., PRKACA, degree 14, and a protein with a low degree, i.e., MRPS5, degree 3. That is, the initial condition vector consists of a vector having one at the entry corresponding to either PRKACA or MRPS5 and zeroes elsewhere. In Fig. 1, we display the changes of the probability with the shortest path distance from the protein where the process starts. This distance corresponds to the number of steps that the perturbation needs to traverse to visit other proteins. For α=1.0, the shapes of the curves in Fig. 1 are the characteristic ones for the Gaussian decay of the probability with distance. However, for α<1, we observe that such decay differs from that typical shape showing a faster initial decay followed by a slower one. In order to observe this effect in a better way, we zoomed the region of distances from 2 to 4 [see Figs. 1(b) and 1(d)]. As can be seen for distances below 3, the curve for α=1.0 is on top of those for α<1, indicating a slower decay of the probability. After this distance, there is an inversion, and the normal diffusion occurs at a much faster rate than the other two for the longer distances. This is a characteristic signature of subdiffusive processes, which starts at much faster rates than a normal diffusive process and then continue at much slower rates. Therefore, here, we observe that the subdiffusive dynamics are much faster at earlier times of the process, which is when the perturbation occurs to close nearest neighbors to the initial point of perturbation.

**FIG. 2.**
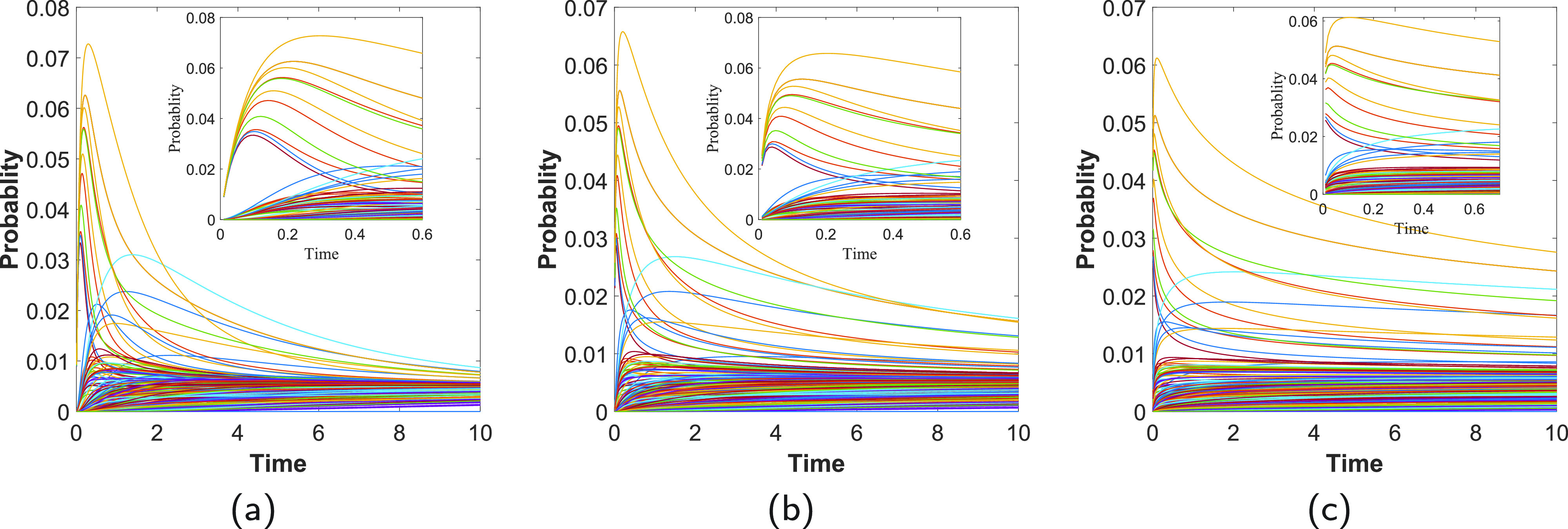
Shock wave increase of perturbations across the network. Time evolution of the propagation of perturbations from the protein PRKACA to the rest of the proteins in the PPI network of human proteins targeted by SARS-CoV-2. (a) Normal diffusion α=1.0. (b) Subdiffusion obtained for α=3/4. (c) Subdiffusion obtained for α=1/2. The insets illustrate the shortest time evolution of the perturbations. Every curve corresponds to a protein in the PPI.

To further investigate these characteristic effects of the subdiffusive dynamics, we study the time evolution of a perturbation occurring at a given protein and its propagation across the whole PPI network. In Fig. 2, we illustrate these results for α=1.0 (a), α=0.75 (b), and α=0.5 (c). As can be seen in the main plots of this figure, the rate of convergence of the processes to the steady state is much faster in the normal diffusion (a) than in the subdiffusive one (b) and (c). However, at very earlier times (see insets in Fig. 2), there is a shock wave increase of the perturbation at a set of nodes. Such kind of shock waves has been previously analyzed in other contexts as a way of propagating effects across PPI networks.^17^ We have explored briefly about the possible causes of this increase in the concentration for a given subset of proteins. Accordingly, it seems that the main reason for this is the connectivity provided by the network of interactions and not a given distribution of the degrees. For instance, we have observed such “shock waves” in networks with normal-like distributions as well as with power-law ones. However, it is possible that the extension and intensity of such effects depend on the degree distribution as well as on other topological factors. The remarkable finding here is, however, the fact that such a shock wave occurs at much earlier times in the subdiffusive regimes than at the normal diffusion. That is, while for α=1.0, these perturbations occur at t≈0.1–0.3; for α=0.75, they occur at t≈0.0–0.2; and for α=0.5, they occur at t≈0.0–0.1. Seeing this phenomenon in the light of what we have observed in the previous paragraph is not strange due to the observation that such processes go at a much faster rate at earlier times, and at short distances, than the normal diffusion. In fact, this is a consequence of the existence of a positive scalar T for which $E_{\alpha ,1}\left(-\gamma t^{\alpha }\right)$ decreases faster than exp⁡(−γt) for t∈(0,T) for $\gamma \in R^{+}$ and $\alpha \in R^{+}$ (see Theorem 4.1 in Ref. 39). Hereafter, we will consider the value of α=0.75 for our experiments due to the fact that it reveals a subdiffusive regime, but the shock waves observed before are not occurring in an almost instantaneous way like when α=0.5 , which would be difficult from a biological perspective.

The previous results put us at a crossroads. First, the subdiffusive processes that are expected due to the crowded nature of the intra-cellular space are very slow for carrying out cellular processes at a significant rate in cells. However, the perturbation shocks occurring at earlier times of these processes are significantly faster than in normal diffusion. To sort out these difficulties, we propose a switching back and restart subdiffusive process occurring in the PPI network. That is, a subdiffusive process starts at a given protein, which is directly perturbed by a protein of SARS-CoV-2. It produces a shock wave increase of the perturbation in close neighbors of that proteins. Then, a second subdiffusive process starts at these newly perturbed proteins, which will perturb their nearest neighbors. The process is repeated until the whole PPI network is perturbed. This kind of “switch and restart processes” has been proposed for engineering consensus protocols in multiagent systems^51^ as a way to accelerate the algorithms using subdiffusive regimes.

### B. Time-fractional diffusion from the SARS-CoV-2 spike protein

The so-called Spike protein (S-protein) of the SARS-CoV-2 interacts with only two proteins in the human hosts, namely, ZDHHC5 and GOLGA7. The first protein, ZDHHC5, is not in the main connected component of the PPI network of SARS-CoV-2 targets. Therefore, we will consider here how a perturbation produced by the interaction of the virus S-protein with GOLGA7 is propagated through the whole PPI network of SARS-CoV-2 targets. GOLGA7 has degree one in this network, and its diffusion is mainly to close neighbors, namely, to proteins separated by two to three edges. When starting the diffusion process at the protein GOLGA7, the main increase in the probability of perturbing another protein is reached for the protein GOLGA3, which increases its probability up to 0.15 at t=0.2, followed by PRKAR2A, with a small increase in its probability, 0.0081. Then, the process switch and restarts at GOLGA3, which mainly triggers the probability of the protein PRKAR2A—a major hub of the network. Once we start the process at PRKAR2A, practically, the whole network is perturbed with probabilities larger than 0.1 for 19 proteins apart from GOLGA3. These proteins are in decreasing order of their probability of being perturbed: AKAP8, PRKAR2B, CEP350, MIB1, CDK5RAP2, CEP135, AKAP9, CEP250, PCNT, CEP43, PDE4DIP, PRKACA, TUB6CP3, TUB6CP2, CEP68, CLIP4, CNTRL, PLEKHA5, and NINL. Notice that the number of proteins perturbed is significantly larger than the degree of the activator, indicating that not only nearest neighbors are activated.

An important criterion for revealing the important role of the protein PRKAR2A as a main propagator in the network of proteins targeted by SARS-CoV-2 is its average diffusion path length. This is the average number of steps that a diffusive process starting at this protein needs to perturb all the proteins in the network. We have calculated this number to be 3.6250, which is only slightly larger than the average (topological) path length, which is 3.5673. That is, in less than four steps, the whole network of proteins is activated by a diffusive process starting at PRKAR2A. Also remarkable that the average shortest diffusive path length is almost identical to the shortest (topological) one. This means that this protein mainly uses shortest (topological) paths in perturbing other proteins in the PPI. In other words, it is highly efficient in conducting such perturbations. We will analyze this characteristics of the PPI of human proteins targeted by SARS-CoV-2 in a further section of this work.

**FIG. 3.**
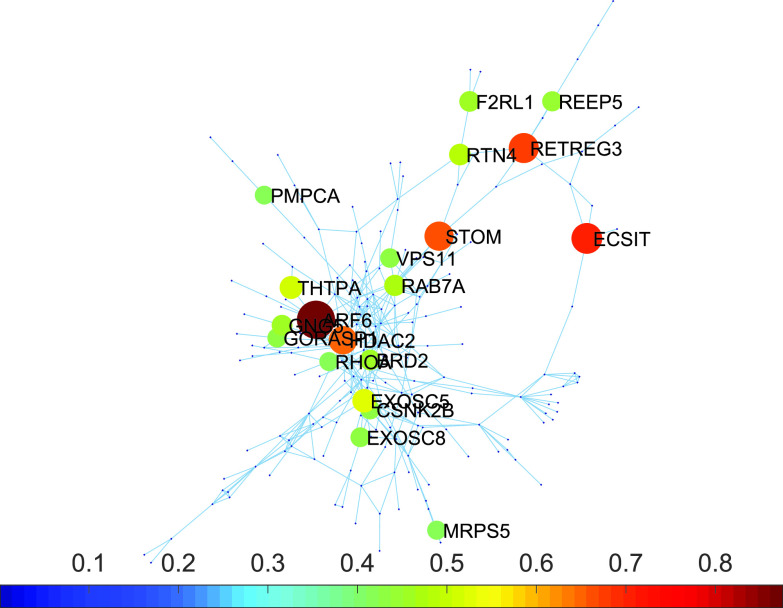
Main disease activators. Proteins targeted by SARS-CoV-2 identified as the top 20 main activators of proteins that are involved in human diseases.

At this time, almost any protein in the PPI network is already perturbed. Therefore, we can switch and restart the subdiffusion from practically any protein at the PPI network. We then investigate which are the proteins with the higher capacity of activating other proteins that are involved in human diseases. Here, we use the database DisGeNet,^52^ which is one of the largest publicly available collections of genes and variants associated with human diseases. We identified 38 proteins targeted by SARS-CoV-2 for which there is a “definitive” or “strong” evidence of being involved in a human disease or syndrome (see Table S1 in the supplementary material). These proteins participate in 70 different human diseases or syndromes as given in Tables S2 and S3 of the supplementary material. We performed an analysis in which a diffusive process starts at any protein of the network, and we calculated the average probability that all the proteins involved in human diseases are then perturbed. For instance, for a subdiffusive process starting at the protein ARF6, we summed up the probabilities that the 38 proteins involved in diseases are perturbed at an early time of the process t=0.2. Then, we obtain a global perturbation probability of 0.874. By repeating this process for every protein as an initiator, we obtained the top disease activators. We have found that none of the 20 top activators is involved itself in any of the human diseases or syndromes considered here. They are, however, proteins that are important not because of their direct involvement in diseases or syndromes but because they propagate perturbations in a very effective way to those directly involved in such diseases/syndromes. Among the top activators, we have found ARF6, ECSIT, RETREG3, STOM, HDAC2, EXOSC5, THTPA, among others shown in Fig. 3, where we illustrate the PPI network of the proteins targeted by SARS-CoV-2 remarking the top 20 disease activators.

### C. Time-fractional diffusion from the lungs to other organs

**FIG. 4.**
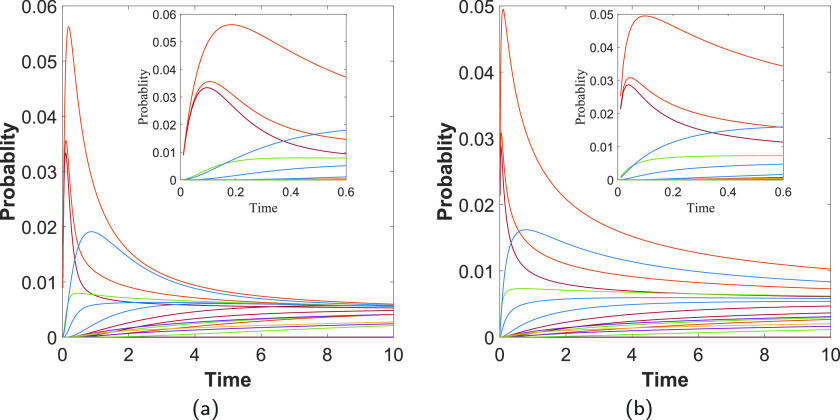
Shock wave increase of perturbations in proteins outside the lungs. Time evolution of the propagation of perturbations from the protein GOLGA2 to proteins mainly expressed outside the lungs. (a) Normal diffusion α=1.0. (b) Subdiffusion with α=3/4. The insets illustrate the shortest time evolution of the perturbation. Every curve corresponds to a protein in the PPI outside the lungs.

We now consider how a perturbation produced by SARS-CoV-2 on a protein mainly expressed in the lungs can be propagated to proteins mainly located in other tissues (see Table S4 in the supplementary material) by a subdiffusive process. That is, we start the subdiffusive process by perturbing a given protein, which is mainly expressed in the lungs. Then, we observe the evolution of the perturbation at every one of the proteins mainly expressed in other tissues. We repeat this process for all the 193 proteins mainly expressed in the lungs. In every case, we record those proteins outside the lungs, which are perturbed at very early times of the subdiffusive process. For instance, in Fig. 4, we illustrate one example in which the initiator is the protein GOLGA2, which triggers a shock wave on proteins RBM41, TL5, and PKP2, which are expressed mainly outside the lungs. We consider such perturbations only if they occur at t<1.

Not every one of the proteins expressed outside the lungs is triggered by such shock waves at a very early time of the diffusion. For instance, proteins MARK1 and SLC27A2 are perturbed in very slow processes and do not produce the characteristic high peaks in the probability at very short times. On the other hand, there are proteins expressed outside the lungs that are triggered by more than one protein from the lungs. The case of GOLGA2 is an example of a protein triggered by three proteins in the lungs. In Table I, we list some of the proteins expressed mainly in tissues outside the lungs, which are heavily perturbed by proteins in the lungs. The complete list of the perturbing proteins is given in Table S5 of the supplementary material. We give three indicators of the importance of the perturbation of these proteins. They are Act., which is the number of proteins in the lungs that activate each of them; $p_{\mathrm{tot}}$, which is the sum of the probabilities of finding the diffusive particle at this protein for diffusive processes that have started in their activators; and $t_{\mathrm{mean}}$, which is the average time required by activators to perturb the corresponding protein. For instance, PKP2 is perturbed by 21 proteins in the lungs, which indicates that this protein, mainly expressed in the heart muscle, has a large chance of being perturbed by diffusive processes starting in proteins mainly located at the lungs. Protein PRIM2 is activated by 5 proteins in the lungs, but if all these proteins were acting at the same time, the probability that PRIM2 is perturbed will be very high, $p_{\mathrm{tot}}\approx 0.536$. Finally, protein TLE5 is perturbed by 13 proteins in the lungs, which needs as an average $t_{\mathrm{mean}}\approx 0.24$ to perturb TLE5. These proteins do not form a connected component among them in the network. The average shortest diffusion path between them is 5.286 with a maximum shortest subdiffusion path of 10. As an average, they are almost equidistant from the rest of the proteins in the network as among themselves. That is, the average shortest subdiffusion path between these proteins expressed outside the lungs and the rest of the proteins in the network is 5.106. Therefore, these proteins can be reached from other proteins outside the lungs in no more than six steps in subdiffusive processes like the ones considered here.

**TABLE I.** Multi-organ propagation of perturbations. Proteins mainly expressed outside the lungs are significantly perturbed during diffusive processes that have started at other proteins expressed in the lungs. Act. is the number of lung proteins activators, $p_{\mathrm{tot}}$ is the sum of the probabilities of finding the diffusive particle at this protein, and $t_{\mathrm{mean}}$ is the average time of activation (see the text for explanations). The tissues of main expression are selected among the ones with the highest Consensus Normalized eXpression (NX) levels by combining the data from the three transcriptomics datasets (HPA, GTEx, and FANTOM5) using the internal normalization pipeline.^49^ Boldface denotes the highest value in each of the columns.

Protein	Act.	$p_{\mathrm{tot}}$	$t_{\mathrm{mean}}$	Tissues of the main expression
PKP2	**21**	0.498	0.29	Heart muscle
CEP43	18	0.521	0.26	Testis
CEP135	14	0.527	0.30	Skeletal muscle, heart muscle, cerebral cortex, cerebellum
TLE5	13	0.509	**0.24**	Thymus, lymph node, testis
RETREG3	6	0.390	0.43	Prostate, thymus
RBM41	6	0.209	0.49	Pancreas, T-cells, testis, retina
PRIM2	5	**0.536**	0.30	Cerebellum, parathyroid gland, testis
MIPOL1	3	0.155	0.57	Pituitary gland, testis
REEP6	1	0.175	0.48	Liver, small intestine, duodenum, testis
HOOK1	1	0.156	0.46	Liver, parathyroid gland, testis, pituitary gland
CENPF	1	0.138	0.46	Thymus, testis, bone marrow
ATP5ME	1	0.096	0.35	Skeletal muscle, dendritic cells, heart muscle, brain
TRIM59	1	0.170	0.69	Corpus callosum, brain, T-cells, testis
MARK1	1	N/A	>100	Epididymis, cerebral cortex, heart muscle, testis, cerebellum
SLC27A2	1	N/A	>100	Liver, kidney

### D. Subdiffusion paths

**FIG. 5.**
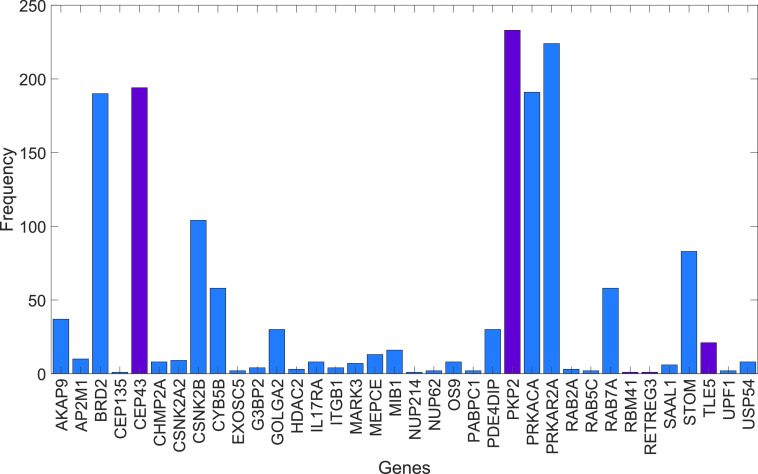
Proteins in shortest subdiffusive paths. The frequency of proteins that appears in the shortest subdiffusion paths but not in the shortest (topological) paths connecting any pair of nodes in the PPI network. Bars marked in wine color are for those proteins expressed mainly outside the lungs.

Finally, we study here how the diffusive process determines the paths that the perturbation follows when diffusing from a protein to another not directly connected to it. The most efficient way of propagating a perturbation between the nodes of a network is through the shortest (topological) paths that connect them. The problem for a (sub)diffusive perturbation propagating between the nodes of a network is that it does not have complete information about the topology of the network as to know its shortest (topological) paths. The network formed by the proteins targeted by SARS-CoV-2 is very sparse, and this indeed facilitates that the perturbations occurs by following the shortest (topological) paths most of the time. Think, for instance, in a tree, which has the lowest possible edge density among all connected networks. In this case, the perturbation will always use the shortest (topological) paths connecting pairs of nodes. However, in the case of the PPI network studied here, a normal diffusive process, i.e., α=1, not always uses the shortest (topological) paths. In this case, there are 1294 pairs of proteins for which the diffusive particle uses a shortest diffusive path, which is one edge longer than the corresponding shortest (topological) path. This represents 6.11% of all total pairs of proteins that are interconnected by a path in the PPI network of proteins targeted by SARS-CoV-2. However, when we have a subdiffusive process, i.e., α=0.75, this number is reduced to 437, which represents only 2.06% of all pairs of proteins. Therefore, the subdiffusion process studied here through the PPI network of proteins targeted by SARS-CoV-2 has an efficiency of 97.9% relative to a process that always uses the shortest (topological) paths in hopping between proteins. In Fig. 5, we illustrate the frequency with which proteins not in the shortest (topological) paths are perturbed as a consequence that they are in the shortest subdiffusive paths between other proteins. For instance, the following is a shortest diffusive path between the two end points: RHOA-PRKACA-PRKAR2A-CEP43-RAB7A-ATP6AP1. The corresponding shortest (topological) path is RHOA-MARK2-AP2M1-RAB7A-ATP6AP1, which is one edge smaller. The proteins PRKACA, PRKAR2A, and CEP43 are those in the diffusive path that are not in the topological one. Repeating this selection process for all the diffusive paths that differs from the topological ones, we obtained the results illustrated in Fig. 5. As can be seen, there are 36 proteins visited by the shortest diffusive paths, which are not visited by the corresponding topological ones. The average degree of these proteins is 7.28, and there is only a small positive trend between the degree of the proteins and the frequency with which they appear in these paths; e.g., the Pearson correlation coefficient is 0.46.

## IV. DISCUSSION

We have presented a methodology that allows the study of diffusive processes in (PPI) networks varying from normal to subdiffusive regimes. Here, we have studied the particular case in which the time-fractional diffusion equation produces a subdiffusive regime, with the use of α=3/4 in the network of human proteins targeted by SARS-CoV-2. A characteristic feature of this PPI network is that the second smallest eigenvalue is very small; i.e., $\mu_{2}=0.0647$. As this eigenvalue determines the rate of convergence to the steady state, the subdiffusive process converges very slowly to that state. What it has been surprising is that even in these conditions of very small convergence to the steady state, there is a very early increase of the probability in those proteins closely connected to the initiator of the diffusive process. That is, in a subdiffusive process on a network, the time at which a perturbation is transmitted from the initiator to any of its nearest neighbors occurs at an earlier time than for the normal diffusion. This is a consequence of the fact that $E_{\alpha ,1}\left(-\gamma t^{\alpha }\right)$ decreases very fast at small values of $t^{\alpha }$, which implies that the perturbation occurring at a protein i at t=0 is transmitted almost instantaneously to the proteins closely connected to i. This effect may be responsible for the explanation about why subdiffusive processes, which are so globally slow, can carry out cellular processes at a significant rate in cells. We have considered here a mechanism consisting in switching and restarting several times during the global cellular process. For instance, a subdiffusive process starting at the protein i perturbs its nearest neighbors at very early times, among which we can find the protein j. Then, a new subdiffusive process can be restarted again at the node j and so on.

One of the important findings of using the current model for the study of the PIN of proteins affected by SARS-CoV-2 is the identification of those proteins that are expressed outside the lungs that can be more efficiently perturbed by those expressed in the lungs (see Table I). For instance, the protein with the largest number of activators, PKP2, appears mainly in the heart muscle. It has been observed that the elevation of cardiac biomarkers is a prominent feature of COVID-19, which in general is associated with a worse prognosis.^53^ Myocardial damage and heart failure are responsible for 40% of death in the Wuhan cohort (see references in Ref. 53). Although the exact mechanism involving the heart injury is not known, the hypothesis of direct myocardial infection by SARS-CoV-2 is a possibility, which acts along or in combination with the increased cardiac stress due to respiratory failure and hypoxemia, and/or with the indirect injury from the systemic inflammatory response.^53–56^ As can be seen in Table I, the testis is the tissue where several of the proteins targeted by SARS-CoV-2 are mainly expressed, e.g., CEP43, TLE5, PRIM2, MIPOL1, REEP6, HOOK1, CENPF, TRIM59, and MARK1. Currently, there is no conclusive evidence about the testis damage by SARS-CoV-2.^57–60^ However, the previous SARS-CoV that appeared in 2003 and which shares 82% of proteins with the current one produced testis damage and spermatogenesis, and it was concluded that orchitis was a complication of that previous SARS disease.^57^ We also detect a few proteins mainly expressed in different brain tissues, such as CEP135, PRIM2, TRIM59, and MARK1. The implication of SARS-CoV-2 and cerebrovascular diseases has been reported, including neurological manifestations as well as cerebrovascular disease, such as ischemic stroke, cerebral venous thrombosis, and cerebral hemorrhage.^61–63^

Kidney damage in SARS-CoV-2 patients has been reported,^64–66^ which includes signs of kidney dysfunctions, proteinuria, hematuria, increased levels of blood urea nitrogen, and increased levels of serum creatinine. As much as 25% of an acute kidney injury has been reported in the clinical setting of SARS-CoV-2 patients. One of the potential mechanisms for kidney damage is the organ crosstalk,^64^ as can be the mechanism of diffusion from proteins in the lungs to proteins in the urinary tract and kidney proposed here. A very interesting observation from Table I is the existence of several proteins expressed mainly in the thymus and T-cells, such as TLE5, RETREG3, RBM41, CENPF, and TRIM59. It has been reported that many of the patients affected by SARS-CoV-2 in Wuhan displayed a significant decrease of T-cells.^67^ Thymus is an organ that displays a progressive decline with age with reduction of the order of 3%–5% a year until approximately 30–40 years of age and of about 1% per year after that age. Consequently, it was proposed that the role of thymus should be taken into account in order to explain why COVID-19 appears to be so mild in children.^67^ The protein TLE5 is also expressed significantly in the lymph nodes. It was found by Feng *et al.*^68^ that SARS-CoV-2 induces lymph follicle depletion, splenic nodule atrophy, histiocyte hyperplasia, and lymphocyte reductions. The proteins HOOK1 and MIPOL1 are significantly expressed in the pituitary gland. There has been some evidence and concerns that COVID-19 may also damage the hypothalamo-pituitary-adrenal axis that has been expressed by Pal,^69^ which may be connected with the participation of the previously mentioned proteins.

Another surprising finding of the current work is the elevated number of subdiffusive shortest paths that coincide with the shortest (topological) paths connecting pairs of proteins in the PPI of human proteins targeted by SARS-CoV-2. This means that the efficiency of the diffusive paths connecting pairs of nodes in this PPI is almost 98% in relation to a hypothetical process that uses the shortest (topological) paths in propagating perturbations between pairs of proteins. The 437 shortest diffusive paths reported here contain one more edge than the corresponding shortest (topological) paths. The proteins appearing in these paths would never be visited in the paths connecting two other proteins if only the shortest (topological) paths were used. What is interesting to note that 6 out of the 15 proteins that are mainly expressed outside the lungs are among the ones “crossed” by these paths. They are TLE5 (thymus, lymph node, testis), PKP2 (heart muscle), CEP135 (skeletal muscle, heart muscle, cerebral cortex, cerebellum), CEP43 (testis), RBM41 (pancreas, T-cells, testis, retina), and RETREG3 (prostate, thymus). This means that the perturbation of these proteins occurs not only through the diffusion from other proteins in the lungs directly to them, but also through some “accidental” diffusive paths between pairs of proteins that are both located in the lungs.

All in all, the use of time-fractional diffusive models to study the propagation of perturbations on PPI networks seems a very promising approach. The model is not only biologically sounded but it also allows us to discover interesting hidden patterns of the interactions between proteins and the propagation of perturbations among them. In the case of the PIN of human proteins targeted by SARS-CoV-2, our current finding may help to understand potential molecular mechanisms for the multi-organs and systemic failures occurring in many patients.

## V. EXPERIMENTAL VALIDATION

After this work was completed, Qiu *et al.*^75^ uploaded the manuscript entitled “Postmortem tissue proteomics reveals the pathogenesis of multiorgan injuries of COVID-19.” The authors profiled the host responses to COVID-19 by means of quantitative proteomics in postmortem samples of tissues in lungs, kidney, liver, intestine, brain, and heart. They reported differentially expressed proteins (DEPs) for these organs as well as virus-host PPIs between 23 virus proteins and 110 interacting DEPs differentially regulated in postmortem lung tissues. According to their results, most DEPs (70.5%) appears in the lungs, followed by kidney (16.5%). Additionally, Qiu *et al.*^75^ identified biological processes that were up- or down-regulated in the six postmortem tissue types. They found that most up-regulated processes in the lungs correspond to processes related to the response to inflammation and to immune response. However, pathways related to cell morphology, such as the establishment of endothelial barriers, were down-regulated in the lungs, which was interpreted as a confirmation that the lungs are the main focus of virus-host fights. Other fundamental processes in the six organs analyzed postmortem were significantly down-regulated, which include processes related to organ movement, respiration, and metabolism.

From the 59 proteins that we reported here as the ones with the largest effect on perturbing those 38 proteins identified in human diseases (see Table S3 in the supplementary material), 18 were found to be down-regulated in the lungs by Qiu *et al.*^75^ If we make the corresponding adjustment, by considering that Qiu *et al.*^75^ considered 110 instead of 209 proteins in the PPI, the previous number represents 58.1% of proteins predicted here and experimentally found as down-regulated in the lungs. From the rest of the proteins, which were not found as having the largest effect on perturbing proteins identified in human disease, only 29.1% were reported by Qiu *et al.*^75^ to be down-regulated in the postmortem analysis of patients’ lungs. Among the proteins reported in Table S3 of the supplementary material and by Qiu *et al.*, we have ARF6, RTN4, RAB7A, 6NG5, REEP5, VPS11, RHOA, RAB5C, among others. Finally, among the proteins mainly expressed outside the lungs that are predicted in this work to be significantly perturbed, we have five that were found by Qiu *et al.*^75^ to be up-regulated in the different organs analyzed by them. From the proteins included in Table I, Qiu *et al.*^75^ reported the following ones up-regulated: PKP2 (heart), REEP6 (liver), HOOK1 (several organs), ATP5ME (heart), and SLC27A2 (liver and kidney). They also reported CEP43 (reported as FGFR1OP) as down-regulated in the brain. We should remark that we have considered here many more organs than the six ones studied by Qiu *et al.*^75^

## VI. FUTURE WORK

There are no doubts that in considering a diffusive propagation of perturbations among proteins in a PPI, we have made a few simplifications and assumptions. Every protein is embedded in an intracellular crowded environment, which drives its diffusive mechanism. Nowadays, it is well-established that this environment is conducive to molecular subdiffusive processes. As remarked by Guigas and Weiss,^27^ far from obstructing cellular processes, subdiffusion increases the probability of finding a nearby target by a given protein, and therefore, it facilitates protein–protein interactions. The current approach can be improved using two recently developed theoretical frameworks: (i) metaplexes and (ii) d-path Laplacian operators on graphs.

**FIG. 6.**
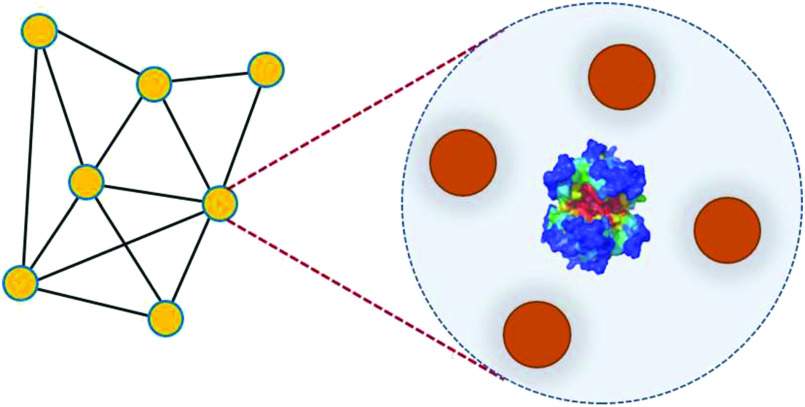
PI metaplex. In the metaplex, every node of the PPI corresponds to a protein and its crowded intracellular space. There is an internal dynamics in the nodes and an external between the nodes.

A PPI metaplex,^70^ a 4-tuple Υ=(V,E,I,ω), where (V,E) is a graph, $\omega ={\left\{\Omega_{j}\right\}}_{j=1}^{k}$ is a set of locally compact metric spaces $\Omega_{j}$ with Borel measures $\mu_{j}$, and I:V→ω is illustrated in Fig. 6. Then, we define a dynamical $\Upsilon =\left(V,E,I,\omega =\{\Omega_{k}\}\right)$ on the metaplex as a tuple (H,T). Here, $H=\{H_{v}:L^{2}\left(\Omega_{I}(v),\mu_{I}(v)\right)\to L^{2}(\Omega_{I}(v),{\mu_{I}(v))\}}_{v\in V}$ is a family of operators such that the initial value problem $\partial_{t}u_{v}=H_{v}(u_{v})$, $u_{v}{|}_{t=0}=u_{0}$, is well-posed, and $T={\left\{T_{vw}\right\}}_{(v,w)\in E}$ is a family of bounded operators $T_{vw}:L^{2}(\Omega_{I(v)},\mu_{I(v)})\to L^{2}(\Omega_{I(w)},\mu_{I(w)})$. This means that inside a node of the metaplex, we consider one protein and its crowded intracellular space. Inside the nodes, we can have a dynamics like a time-fractional diffusion equation, the fractional Fokker–Planck equation, or any other in a continuous space. The inter-nodes dynamics is then dominated by a graph-theoretic diffusive model like the one presented here.

The second possible improvement to the current model can be made by introducing the possibility of long-range interactions in the inter-nodes dynamics in the PPI metaplex. That is, instead of considering the time-fractional diffusion equation, which only accounts for subdiffusive processes in the graph, we can use the following generalization, which incorporates the d-path Laplacian operators,^71^

$$D_{t}^{\alpha }x\left(t\right)=-C\left(\sum_{d=1}^{diam}d^{-s}L_{d}\right)x\left(t\right),$$ (23)

where $L_{d}$ is a generalization of the graph Laplacian operator to account for long-range hops between nodes in a graph, d is the shortest path distance between two nodes, and s>0 is a parameter. This equation has never been used before except that for the case α=1 where superdiffusive behavior was proved in 1- and 2-dimensional cases.^72,73^ Other approaches have also been recently used for similar purposes in the literature.^74^

We then hope that the combination of metaplexes and time- and space-fractional diffusive models do capture more of the details of protein–protein interactions in crowded cellular environments.

## SUPPLEMENTARY MATERIAL

See the supplementary material for a list of proteins targeted by SARS-CoV-2, which are found in the database DisGeNet as displaying “definitive” or “strong” evidence of participating in human diseases. The Disease ID is the code of the disease in DisGeNet. A list of proteins with the largest effect on perturbing those 38 proteins are identified in human diseases. $p_{tot}$ is the sum of the probabilities that the given protein activates those identified as having “definitive” or “strong” evidence of being involved in a human disease. There is an RNA expression overview for proteins targeted by SARS-CoV-2 and mainly expressed outside the lungs. We select the top RNA expressions in the four databases reported in The Human Protein Atlas. There is a list of proteins mainly expressed outside the lungs and their major activators, which are proteins mainly expressed in the lungs.

## Data Availability

The data that support the findings of this study are available within the article and its supplementary material and also from the corresponding author upon reasonable request.
